# Obesity and understudied minority children: existing challenges and opportunities in epidemiology

**DOI:** 10.1186/s12887-019-1484-9

**Published:** 2019-04-10

**Authors:** Zerleen S. Quader, Julie A. Gazmararian, Lauren E. McCullough

**Affiliations:** 1 Department of Epidemiology, Emory University, CNR 3rd floor, 1518 Clifton Road, NE, Atlanta, GA 30322 USA; 2 Winship Cancer Institute, Emory University, Atlanta, GA USA

**Keywords:** Obesity, Children, Adolescents, Race-ethnicity

## Abstract

**Background:**

Obesity is a major public health concern in the United States and should be addressed as early as possible, in childhood. Disparities exist in obesity prevalence and its associated comorbidities by racial/ethnic group, however less is known about the smaller racial/ethnic subclasses that are often aggregated and assumed to be homogeneously at risk. As the racial and ethnic composition of the US shifts towards greater diversity, it is important that epidemiologic research addresses these new challenges.

**Main body:**

In this short communication, we focus on Asian American children given that subgroups are historically understudied and emerging evidence among adults suggest heterogeneous associations for both obesity and cardio-metabolic outcomes. Existing limitations in this research area include: (1) identifying the appropriate measurement of adiposity in Asian American children; (2) determining high-risk cutoffs for intervention; and (3) developing strategies to ensure study robustness.

**Conclusion:**

Data disaggregation is a necessary approach to understand potentially heterogeneous associations in childhood obesity and cardio-metabolic risk, but epidemiologic investigators must address these challenges. Ultimately, successful strategies could help better identify high risk subgroups, target interventions, and effectively reduce the burden of obesity among American youth.

## Background

In the United States (US) almost a third of children aged 2–19 years are overweight or obese, and the presence of obesity in childhood is associated with development of risk factors for cardiovascular disease [1]. While obesity is largely prevalent in the US, it does not affect all population subgroups equally. Disparities in obesity and associated cardio-metabolic risk often begin in childhood and persist into adulthood, highlighting the importance of addressing these health concerns in early life [2]. While adiposity and fat deposition may differ by race/ethnicity, epidemiologic research examining disparities in obesity risk among children has largely focused on general racial/ethnic groupings, wherein ethnic subgroups are often categorized as a single homogenous group. For example, Hispanic subgroups such Mexican, Central American, South American (among others) are often aggregated into one; differences in health status by country of origin among African Americans has been noted; and “Asian American” is often used in epidemiologic studies, rather than more descriptive groups such as Vietnamese American or Chinese American.

Potential variability in cardio-metabolic risk associated with overweight/obesity is unresolved when these groups are assumed homogeneous. Addressing these potential differences among racial and ethnic minority children in the US is important for public health practice and research. Census projections suggest that over the next four decades, a larger proportion of the US population will be foreign born, and the proportion of minorities in the US is expected to continue to grow, increasing diversity in the US [3]. By 2044 it is expected that no one racial/ethnic group will compose more than 50% of the population [3]. Asian Americans are the fastest growing ethnic group in the US, having grown by 46% between 2000 and 2010 compared to a 10% increase in the total population [4]. Within the Asian population, detailed ethnic groups varied in their rate of increase between 2000 and 2010. Chinese, Asian Indian, and Filipino populations are the three largest Asian subgroups in the US, and increased by 40, 68, and 45% respectively. Other groups, such as Pakistani or Nepalese, which make up much smaller portions of the Asian population increased by at least 100% and many small groups much more than that [4]. Thus, a more granular understanding of associations with obesity and cardio-metabolic disease in these subgroups may be informative for public health messaging and intervention. While global challenges exist in understanding childhood obesity as it relates to downstream health outcomes, including: (1) difficulties in measurement; (2) appropriateness of cut points for risk stratification; and (3) power to estimate stratum specific effects by ethnic subgroup, we consider these challenges as they specifically relate to Asian American youth.

## Main text

### Measuring adiposity in Asian-American children has unique challenges

An existing issue with addressing the role of childhood obesity in early and late health outcomes is the difficulty in measurement of adiposity. There is significant variability in the measurements used to ascertain overweight and obesity status among children and adolescents. Often, the appropriate measurement depends on the goals of research (e.g., clinical and diagnostic importance versus population-based research). Body mass index (BMI kg/m^2^) is most commonly used, however its diagnostic ability has been shown to vary considerably [5], and BMI percentiles for age and sex, used for research in children, may not reflect body fatness equally well across racial/ethnic groups [5]. BMI does not distinguish between fat and fat-free mass and cannot identify central adiposity. Other metrics, such as waist-to-height ratio and waist circumference, have proven valuable in linking adiposity to chronic conditions such as metabolic syndrome and diabetes in adults [6], and may represent a more sensitive measure in certain population subgroups [7]. However, it remains unresolved whether these metrics are equally useful for predicting obesity, and downstream chronic disease, in children and adolescents [8].

Measurement of adiposity can be particularly challenging in studies of racial/ethnic minorities due to differences in fat distribution across subgroups that are missed when subgroups are aggregated. For example, among Asian Americans, Filipino adults typically have higher levels of visceral fat, yet in this subgroup visceral adipose tissue is not as strongly correlated with BMI as compared to white adults [9]. South Asians have also shown greater levels of visceral adipose tissue, and in parallel, both of these groups have a higher risk of type 2 diabetes [10]. Variability in fat patterning between groups is likely genetic, and therefore may emerge in childhood. Differences in fat distribution have been observed in Asian American, white, and black prepubertal children, and BMI trajectories from adolescence into adulthood are known to vary by race/ethnicity [2, 11]. Capturing this variability in a diverse group such as Asian American children can provide an opportunity to understand how to better utilize anthropometric indices for obesity research and practice.

### High-risk cut points are poorly defined and lack specificity

Given that excess body weight is a risk factor for many chronic diseases, risk stratification is important for prevention, screening, diagnosis, and management of cardio-metabolic conditions. Childhood obesity has been shown to be associated with markers of atherosclerosis, hypertension, and insulin resistance, among several other cardio-metabolic risk factors [12]. Although BMI is commonly used for risk classification, established cut points may have lower sensitivity to cardio-metabolic risk factors in certain population subgroups [12]. Even within defined weight classifications, risk levels can differ depending on genetics, fat distribution, and body type.

While this issue may also be present for children in larger racial/ethnic groups, this is clearly exemplified in investigations focused on adult Asian American subgroups where significant heterogeneity in overweight, obesity, and diabetes prevalence has been observed [13]. For the same BMI level, Asian adults may have an increased risk of cardio-metabolic risk factors, leading the World Health Organization to suggest separate cut points to assess overweight/obesity status for this ethnic group [14]. The American Diabetes Association made a similar recommendation, that testing for diabetes should be considered for all Asian American adults with a BMI greater than or equal to 23 kg/m^2^, lower than the 25 kg/m^2^ that is considered overweight [15]. However, despite this recognized heterogeneity among Asian adults, limited data are available on the association between early-life obesity and early cardio-metabolic risk among Asian American children. Evidence suggests that Asian adolescents who are overweight may be at higher risk for insulin resistance compared to their non-Asian counterparts of the same weight, and these associations may vary markedly within subpopulations of Asian Americans [16]. Efforts to characterize the heterogeneity in risk among Asian American youth are needed.

### Availability of data

Data around appropriate measurements and meaningful classification for adiposity and cardio metabolic risk in subgroups of racial and ethnic minorities are only beginning to emerge among adults and are relatively absent in the childhood literature. Among Asian Americans, few studies report strata-specific associations or prevalence of overweight/obesity among Asian American youth, and those that do rely largely on data from population-based studies that oversampled Asian-Americans (Table 1). To date, only one study had Asian subgroup specific sample sizes greater than 200, and is limited to California [17]. Moreover, there are no longitudinal data of obesity and subsequent cardio-metabolic risk factors in Asian-American children/adolescents. A critical barrier to addressing these challenges, is obtaining robust quality data across racial/ethnic subgroups of children. Data disaggregation has not been fully endorsed because of challenges such as difficulties with data collection, participant recruitment, and lack of consistency in definitions of ethnic subgroups across studies [18]. However, such approaches may be necessary as population demographic distributions in the US shift.

**Table 1:** Reported % overweight/obese among Asian American children, by subgroup

Population Source	Jain, 2012 [19]		Guerrero, 2015 [17]		Diep, 2017 ^a^ [20]		APIAHF ^b ^[21]	
	Children aged 4; Early Childhood Longitudinal Study (ECLS)–Birth Cohort, 2001		Children aged 2–11; California Health Interview Surveys, 2007–09 & 2011–12		Children attending kindergarten- 2nd grade; Early Childhood Longitudinal Study –Kindergarten Class, 2010–11		Children/adolescents aged 2–19; National Health and Nutrition Examination Survey, 2011–14	
	N	(%)	N	(%)	N	(%)	N	(%)
Chinese	400	(23.5)	717	(27.8)	300	(11.2)	185	(11.8)
Japanese	50	(24.0)	227	(42.1)	–		91	(18.1)
Filipino	150	(28.4)	455	(39.6)	100	(24.8)	111	(29.5)
Asian India/South Asian	100	(15.6)	403	(27.1)	350	(17.9)	256	(18.4)
Korean	50	(20.2)	385	(26.8)	–		--^c^	
Vietnamese	50	(34.7)	625	(34.1)	150	(16.2)	153	(27.3)
Other	300	(29.8)	390	(35.5)	300	(17.1)	–	

^a^% consistently overweight/obese

^b^Asian & Pacific Islander American Health Forum

^c^Japanese/Korean combined into one group

## Opportunities

While several challenges to addressing childhood obesity epidemiology in minority populations have been enumerated above, there are many opportunities for future research, specifically:

- Validation of measures of adiposity in classifying obesity in ethnic subgroups of children/adolescents to identify if, and at what ages, different cut points are warranted;
- Examination of trajectories of weight status/BMI among different subgroups to identify early differentiation;
- Exploration of potential early life, pregnancy, and in-utero variation that may account for differences in cardio-metabolic risk factors; and
- Additional exploration into the role of nativity and immigration status.

In addition to filling evidence gaps, researchers interested in exploring disparities and differences between subgroups can consider:

- Active communication and collaboration with communities of interest;
- Increasing efforts to oversample populations in regularly collected surveillance data, particularly as the US becomes increasingly diverse; and
- Pooling resources to increase study power and preserve anonymity of participants in larger prospective studies.

## Conclusion

Although challenges exist in conducting epidemiologic research among subgroups of racial/ethnic minority children, focusing on the existing heterogeneity in this population introduces opportunities to explore potential mechanisms through which ethnicity is associated with childhood obesity. Additional understanding of contributors to obesity, both environmental and biological, could eventually translate into public health programs and interventions for high-risk groups or areas. Asian American youth represent a unique opportunity to study the effects of immigration, acculturation, and genetic influences on obesity and its comorbidities. Researchers aimed at exploring childhood obesity in diverse populations should oversample ethnic subgroups and consider large longitudinal studies of diverse youth. Continued research among understudied minority groups is important to address challenges in measurement and risk assessment, and introduces opportunities to identify unique ways to modify risk for obesity that can be translated into public health practice.

## Abbreviation

BMI: Body Mass Index
